# The Cultural Importance of Plants in Western African Religions

**DOI:** 10.1007/s12231-018-9410-x

**Published:** 2018-04-03

**Authors:** Diana Quiroz, Tinde van Andel

**Affiliations:** 1 Hamburg University (Biocentre Klein Flottbek and Botanical Garden), Ohnhorststr. 18, 22609 Hamburg, Germany; 2 Universidad Nacional Autónoma de México (Instituto de Investigaciones en Ecosistemas y Sustentabilidad), 58190 Morelia, Michoacán Mexico; 3 Naturalis Biodiversity Center, P.O. Box 9517, 2300 RA Leiden, The Netherlands; 4 Wageningen University, Biosystematics Group, P.O. Box 647, 6700 AP Wageningen, The Netherlands

**Keywords:** Benin, Bwiti, cultural domain analysis, Gabon, ethnobotany, folk religions, Vodoun

## Abstract

This paper explores the importance of elements of the natural world, and particularly plants, among people of different religious affiliations in western Africa*.* Plants play an overriding role in African folk religions, which in turn are closely associated with health practices and influence management decisions concerning natural resources. In spite of the extensive literature documenting ritual plant use, the cultural importance of plants in this context has not been systematically assessed. Our objective was to see whether the importance of plants was reflected in people’s conceptions of *global* (i.e., Christianity, Islam) and *folk* religions (i.e., Vodoun and Bwiti) in Benin (West Africa) and Gabon (Central Africa). By performing a cultural domain analysis (CDA) with 96 individuals, we found that, regardless of the religious affiliation of informants, plants and other elements of the natural world were more present in people’s notions of folk religions than in global religions. We conclude by reflecting on the potentials and limitations of the data presented here as a starting point to explore the topic of cultural keystone species.

**Electronic supplementary material:**

The online version of this article (10.1007/s12231-018-9410-x) contains supplementary material, which is available to authorized users.

## Introduction

Researchers have tended to label the unknown or the incomprehensible in other cultures as religious or mystical (Bowie 2008). In the early history of anthropology, these epithets often had a pejorative connotation. The paradigm shift brought by functionalism in anthropology had pivotal repercussions for different fields of research (Gould 1966). In the case of ethnobotany, for instance, plants that were once considered “primitive” (Chevalier 1937: 94) and with “plain mumbo-jumbo uses” (Burkill 1985: xiii) are now recognized for their importance in both local healthcare practices (Coks and Moller 2002; Janzen and Green 2003; Mafimisebi and Oguntade 2010; Quiroz et al. 2016; van Andel and Ruysschaert 2011), as well as for the crucial role they play in management decisions regarding the use of natural resources and the preservation of biodiversity (Msuya and Kindeghesho 2009; Quiroz and van Andel 2015). While the term has been a recurrent topic in ethnobotanical documentation in the past century, “religion” appears to lack a unified definition in the field. Probably due to the elusiveness of the term, which lacks an approximate translation in non-western languages (Bowie 2008), terms such as “medico-magic,” “magico-religious,” “sacred,” “(spi) ritual,” “supernatural,” “mystical,” and “magic” all appear to form part of the domain religion in the considerable number of publications that address plant use in contexts that involve supernatural agents (Albuquerque et al. 2007; Cavender and Albán 2009; de Souza 2006; Mafimisebi and Oguntade 2010; Robson et al. 1982; Sharma et al. 2012; van Andel et al. 2012; Voeks 1993, 1997).

Agency is one of the central concepts in studies that address the relationships between humans and nature (Moran 2006). It is understood as the capacity of an entity to act in the world. Agents can be human, non-human, physical, or non-physical. While human agents are acknowledged for their primacy in the co-evolutionary process that leads to environmental change (Bandura 2006), questions have been raised about the actual agency exercised by non-human and non-physical entities (Nash 2005). This point of view is substantiated by the fact that (1) non-physical agents such as spirits and gods simply cannot directly act in physical ways and (2) non-human agents (e.g., animals, plants, and objects) lack the essential attributes that characterize agency. These are intentionality, foresight, and self-reflectiveness of action and its consequences (Bandura 2006; Nash 2005). What is known about non-human and non-physical agents, however, is that their agency is enacted through human agents (Robinson 2011), as evidenced, for instance, by the wealth of archeological documentation on the material correlates of animism (Brown and Walker 2008). In our work, we have acknowledged non-human and non-physical entities for their agency as the underlying forces through which humans manage plants in the context of traditional religious practices.

We performed a Cultural Domain Analysis, a method commonly used in cognitive anthropology and marketing research for the purpose of highlighting the underlying properties of a given cultural domain from the viewpoint of informants (Borgatti 1994). Our objectives were to understand the cultural domains *global* and *folk* religions from an emic perspective, and to explore the importance of plants within these two domains in two African countries: Benin and Gabon.

In Benin, folk religions are recognized as official religions. Vodoun or Orisha, the most prevalent traditional faith in the country, is based on the belief of supernatural gods that help the creator govern the natural world (Herskovits 1938). A wide variety of ritual plants are sold at Benin’s urban markets (Quiroz et al. 2014). In Gabon, Bwiti is a social and religious institution comprised by secret societies, each with its own passage rites and ceremonies (Świderiski 1965). Albeit not officially recognized, folk religions are practiced or at least tolerated by a considerable segment of the Gabonese population (US Department of the State 2012). Just as in Benin, plants play a central role in Bwiti practices (Raponda-Walker and Sillans 1962) and are commercialized in substantial quantities on the Gabonese domestic market (Towns et al. 2014).

We wanted to assess whether plants and other elements of the natural world were more often present in the informants’ idea of folk religions than in their notion of global religions. We also wanted to know whether these differences would show when making comparisons according to the ethnic and religious backgrounds of the informants. We acknowledged the possibility that the term religion, and the notions that its use elicited, would carry an ethnocentric bias based on Western thought and values (Dubuisson 2003). Therefore, we hypothesized that informants would hardly mention plants, or other elements of the natural world, when asked to list the names of categories that belonged to the domain global religion. We also expected that for people who considered themselves followers of Bwiti or Vodoun, plants would more obviously form part of their conception of folk religion than for non-followers of these religions.

### Study Sites

**Fig. 1:**
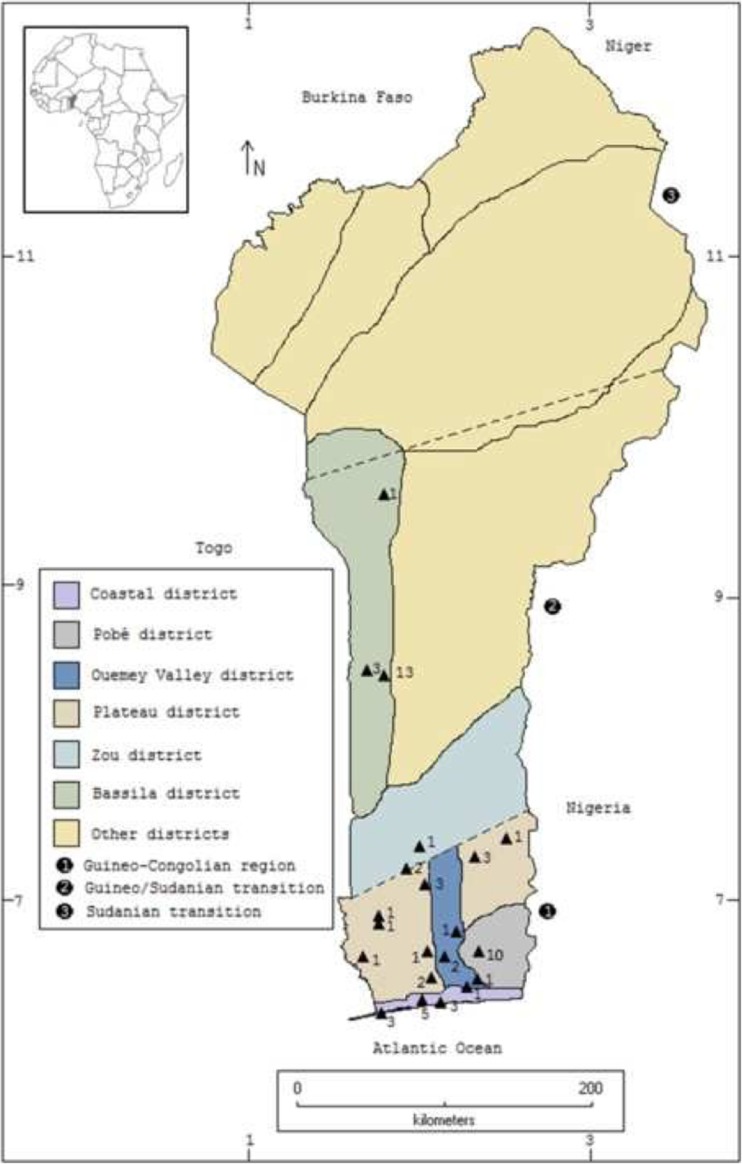
Fieldwork locations in Benin. Triangles indicate surveyed locations. Numbers indicate informants per location. Source Quiroz and van Andel (2015).

**Fig. 2:**
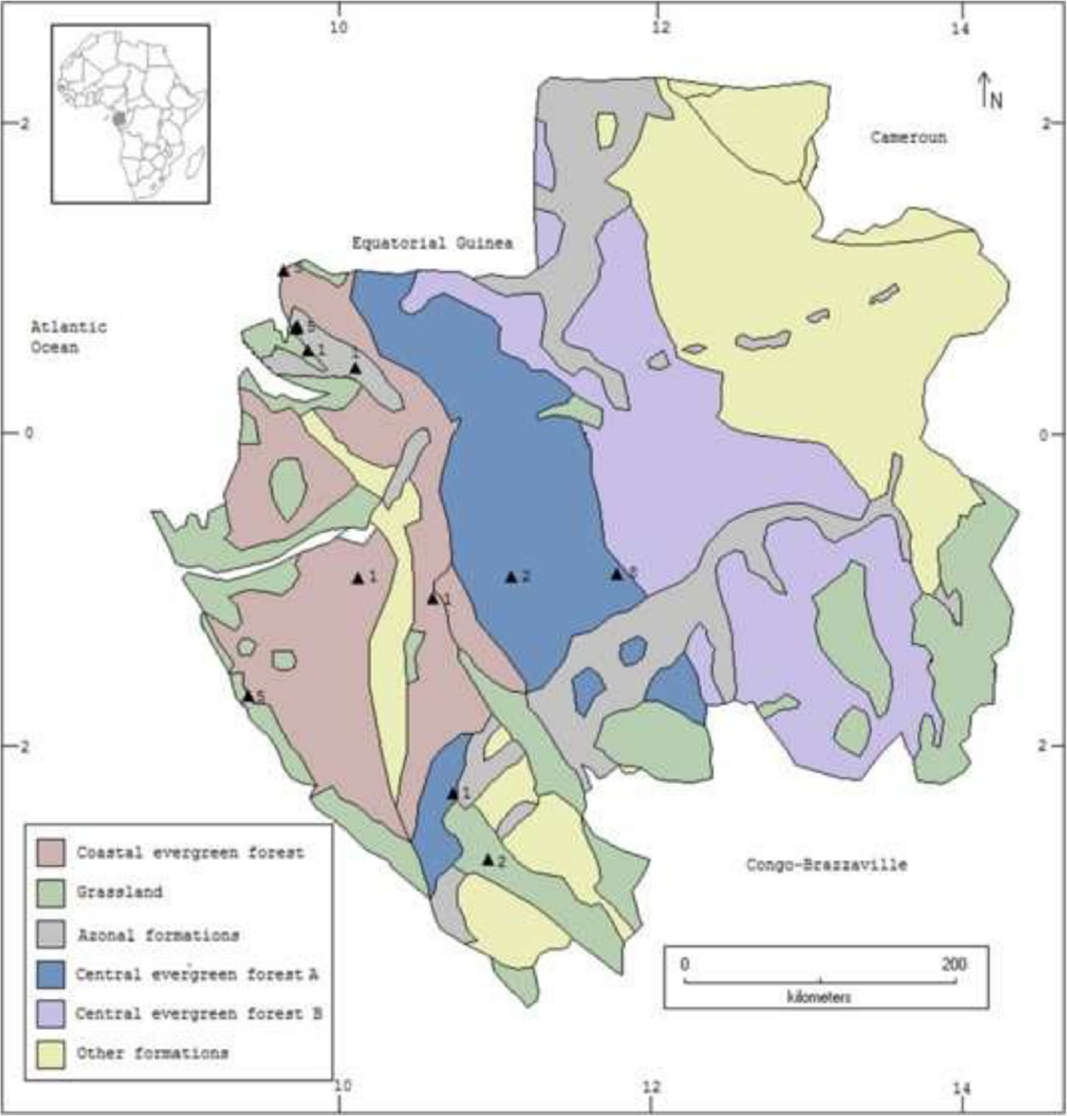
Fieldwork locations in Gabon. Triangles indicate surveyed locations. Numbers indicate informants per location. Source Quiroz and van Andel (2015).

This study was carried out in different locations across Benin, West Africa, and Gabon, Central Africa. Benin is located in the Dahomey Gap, the corridor of savanna vegetation that separates the Upper and Lower Guinea forest (Fig. 1). Gabon is located in the latter (Fig. 2). Apart from the dissimilar biogeography, substantial cultural differences exist between the two countries. Linguistically, the peoples of Benin included in this study belong to the Ewe and Yoruba, and in Gabon to the Bantu-Kikongo groups (William and Blench 2000). Access to primary education is widespread in both countries, resulting in the command of French by large sectors of the population. It is not uncommon, however, to find older individuals and women who only speak the local languages, especially in rural areas. In Gabon’s capital Libreville, many younger inhabitants speak only French. The flourishing and social acceptance of two well-known folk religions in these two countries facilitated our choice for comparison.

## Methods

### Data Collection

We conducted fieldwork in Benin in 2011 and in Gabon in 2012. We used a multiple purposive technique (Teddlie and Yu 2007) in order to obtain a sample of 99 informants in the two countries. This method involved a combination of convenience and chain-referral sampling (Penrod et al. 2003). As the work presented in this paper formed part of a larger scientific study on ritual plant use in Benin and Gabon (Quiroz 2015), in cooperation with local universities, we recruited the majority of the participants among students, market vendors, and traditional healers. We obtained data on the informants’ socio-demographic background and paid special attention to recording their religious affiliation (i.e., whether they considered themselves followers of folk religions or not). Due to the conflictive nature of admitted affiliation to folk religions (Bonhomme 2007; Chenry 2008) and in order to avoid possible under-reporting, we did not ask informants for their names.

We conducted free-listing exercises for the purpose of recording the items that, from the viewpoint of the informants, formed the cultural domains “global religion” (*la religion*, in French) and “folk religions,” for which we used the term *Vodoun* in Benin and *Bwiti* in Gabon. Following Puri (2011), informants were given 2 min to execute the exercise and completed the free lists themselves in written form. In case participants were not able to read and write, the exercise was performed orally and recorded on audiotape. All exercises were conducted individually. Informants were compensated for their time with a small sum of money that was convened with them prior to the free-listing exercise.

### Data Analysis

Following Puri (2011), we elaborated a matrix for each domain with the items mentioned by the informants ordered as rows. We used codes for the individual informants and included them in the matrix as columns. We entered the rank numbers of items as they occurred on each of the informants’ lists (with rank number 1 as most important), adding new items as successive rows. Individual plant names and organs derived from a single species were documented, but for the purpose of analysis merged with general plant organs (e.g., bark or leaves) or populations (expressed as trees or forests) under the broader category “plants.” We did, however, consider individual plant species in our qualitative discussions. We verified the identity of all plant species mentioned in the free-listings by checking their vernacular names against our database of botanical collections (Quiroz 2015), collected over a 1-year period of fieldwork in Benin and Gabon and containing 618 botanical species with 667 ritual uses and 688 vernacular names. Other terms inherent to the cultures of our study countries that were unknown to us were checked with key informants (i.e., elder traditional healers) during informal interviews or by consulting anthropological literature on Benin and Gabon (Herskovits 1938; Raponda-Walker and Sillans 1962). All terms mentioned during the interviews are listed, when necessary with a brief explanation of their meanings, in the Electronic Supplementary Material (ESM).

Next, we created separate presence-absence matrices by substituting the ranks given to each term in the free-list with the number one. We conducted a Detrended Correspondence Analysis (DCA) to define informant groups and to identify the two main axes that caused the distribution of terms in the cultural domain space. In order to reduce axis length, we down-weighed rare terms. We plotted the first and second axes in a two-dimensional graph to examine the potential overlap among informants and to visualize the variation within and between the two countries. To see whether plants appeared more often in the cultural domain “folk religions” than in “global religion” and to see whether this differed among people of various cultural backgrounds or religious affiliations, we calculated the frequency (number of informants mentioning a term divided by the total number of informants) and average ranks of all terms mentioned by each group that had resulted from the DCA, and plotted them to visualize their saliency, as described by Puri (2011). Because of the large number of items in the free-lists, we only considered terms with frequencies higher than 0.10.

## Results and Discussion

### “Global Religion” Vs. “Folk Religions”

From our initial sample of 99 informants, three were excluded because a lack of socio-demographic data (Table 1). Over half of the informants (59%) self-reported adherence to global religions, most of them as Christians (56%), while 41% were said to follow folk religions in both countries. Due to time constraints, the number of informants in Gabon was half as small as in Benin (Table 1). With the combined responses of both followers and non-followers in Benin and Gabon for the cultural domains “global religion” and “folk religions,” a list of terms including 425 items was compiled (ESM). From those 425 terms, 86 (about 20% of the total) formed part of both cultural domains—global religion and folk religion.

**Table 1:** Socio-demographic characteristics of 96 participants.

Characteristic	*N*	%
Country		
Benin	66	69
Gabon	30	31
Age (year)		
< 20	6	6
20–29	41	42
30–39	22	23
40–49	12	13
50–59	8	9
> 60	7	7
Gender		
Male	52	54
Female	44	46
Religious affiliation		
Catholic	11	12
Protestant	4	4
Other Christian	39	40
Muslim	3	3
Vodoun	20	21
Bwiti	19	20
Occupation		
Student	38	39
Traditional healer	14	15
Market vendor	32	33
Other	12	13

**Fig. 3:**
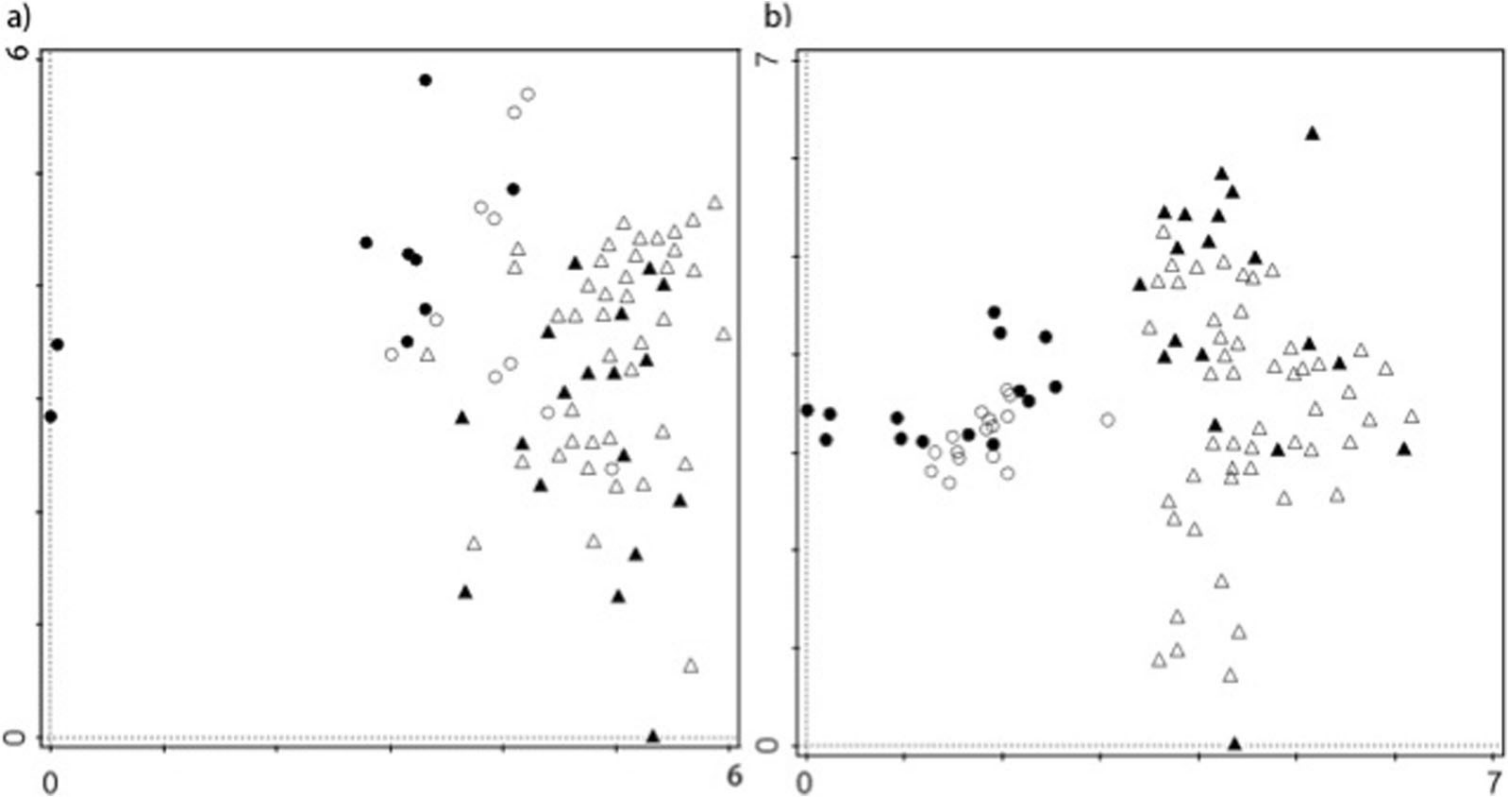
DCA scatterplots comparing followers (black) and non-followers (white) of traditional religions in Gabon (circles) and Benin (triangles) for the domains a) *global religion* and b) *folk religions*.

The results of the DCA (Fig. 3a) show that the informants’ notions of global religion did not differ much across religious affiliation or country of origin. The free list for this category was composed of 206 terms with 184 terms provided by all Beninese informants and 39 by the Gabonese ones. Sixteen items (around 8% of the total) were provided in both countries, including words such as “God,” “Bible,” “church,” and “faith.” On average, each informant mentioned 9.3 terms in Benin and 3.0 in Gabon. Followers of folk religions from both countries provided an average of 5.6 words, whereas non-followers mentioned 6.8.

The low number of terms provided by the Gabonese informants for global religion is remarkable. The online dictionary of Bantu languages (http://www.cbold.ish-lyon.cnrs.fr) has no record of a term for religion in the Nzebi, Fang, Punu, and Mpongwe languages. This could explain the short lists provided by informants in the Gabonese free lists., even though these exercises were executed in French and they understood the term “la religion.”

With a few informants, however (all of them from Babongo ethnicity, otherwise known as Pygmies), we could only conduct our work with the assistance of an interpreter due to their insufficient knowledge of French. Probably, the term “la religion” did not have an equivalent in the Babongo language either, as those informants provided no items for the term “global religion” and none of them reported adherence to either Christian, Islamic, or other global religions.

**Fig. 4:**
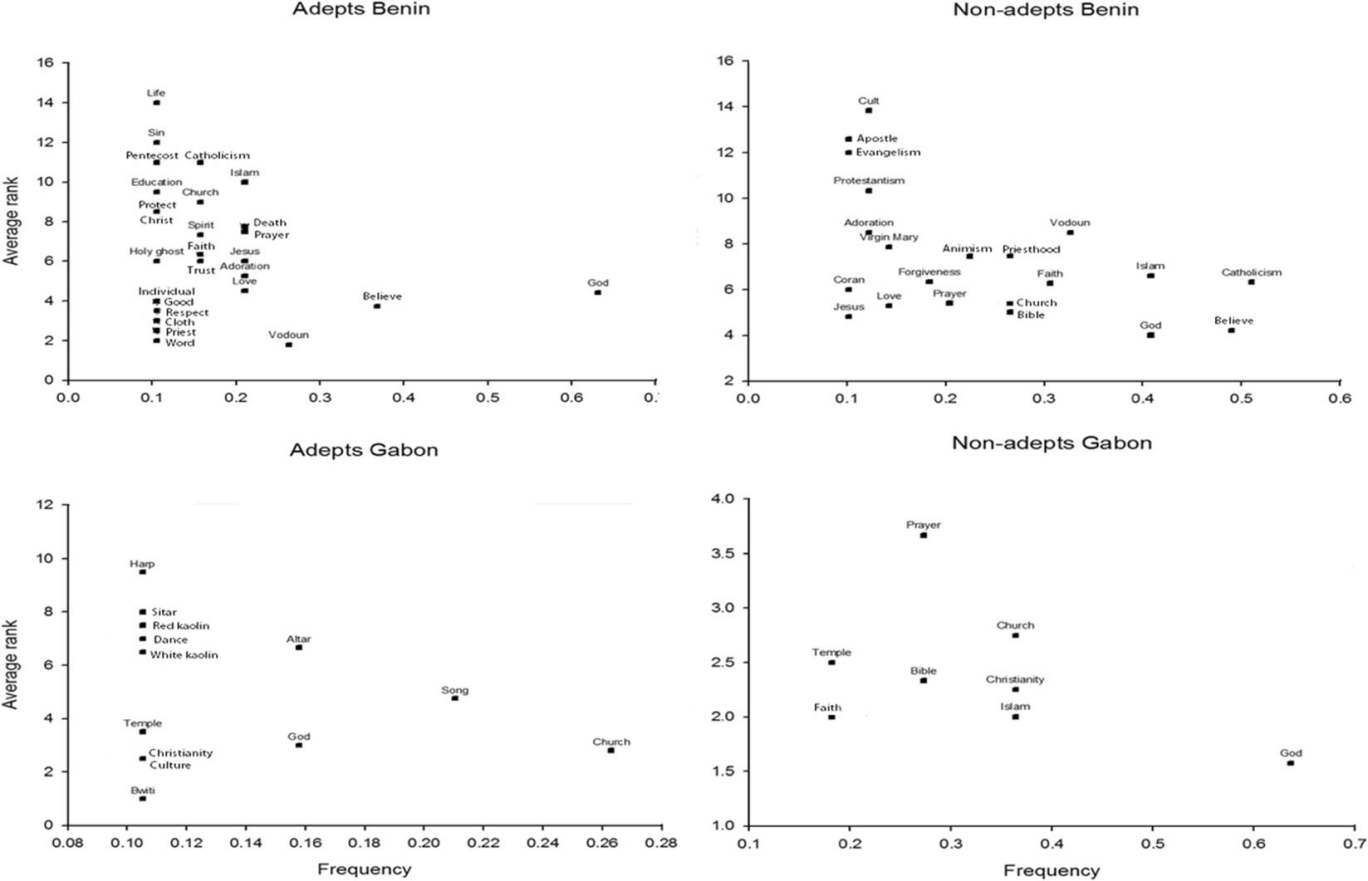
Scatterplot of terms with frequency > 0.10 for responses provided by all four different informant groups for the domain *global religion*. The higher the frequency and the lower average rank of a term, the higher its saliency.

From the scatter plot of terms (Fig. 4), it is clear that within the cultural domain religion, all informant groups mentioned items of Abrahamic religions (e.g., God, “Islam,” “Catholicism,” church, and Bible). However, with the exception of non-followers of traditional faiths in Gabon, some informants in the other three groups also mentioned aspects of folk religious practices when asked to define religion. For instance, informants considered the terms “Vodoun” in Benin, and “kaolin” (white clay and red pigments used in traditional ceremonies) and traditional musical instruments played during ceremonies (“harp,” “sitar,” “drums”) in Gabon as part of *global religion*. The terms “medicinal plants” and “leaves” (*les feuilles* in French, which locally also means herbal medicine) also formed part of *global religion* for some followers of Vodoun in Benin, but their rank was very low (ESM). We therefore accept our hypothesis that plants are not generally associated with the domain *global religion*.

In the case of the cultural domains “Vodoun” and “Bwiti,” the results from the DCA showed a trend influenced by the religious affiliation (i.e., followers and non-followers) and country of origin of the informants. Beninese and Gabonese informants formed two clearly defined groups, with followers of folk religions generally segregated from non-followers (Fig. 3b). In total, 300 terms were named by all informants for this category (238 in Benin and 100 in Gabon), while 38 items (roughly 13% of the total) were mentioned in both countries. On average, each informant mentioned 11.1 items in Benin and 11.7 in Gabon. Followers of folk religions in both countries provided an average of 12.3 words, whereas non-followers mentioned 9.5.

### The Cultural Domain “Vodoun”

**Fig. 5:**
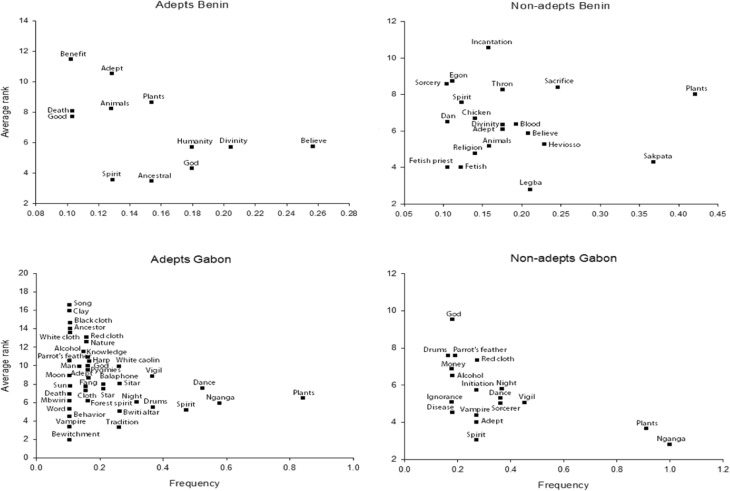
Scatterplot of terms with frequency > 0.10 for responses provided by all four different informant groups for the domain *folk religions*, defined as “Vodoun” in Benin and “Bwiti” in Gabon. The higher the frequency and the lower average rank of a term, the higher its saliency.

Although “plants” was a term highly cited by all Beninese informants, on the sole basis of its saliency (i.e., citation frequency and average rank), it was more closely associated to Vodoun by non-followers than by followers of folk religions (Fig. 5). We obtained similar results in the number of responses by the different groups that included elements of the natural world. Non-followers mentioned three times as many words of this type than the followers of folk religions. Terms such as “earth,” “water,” “air,” and “lightning” formed part of the non-followers’ conception of folk religions. Thus, for Benin, we reject our second hypothesis that for followers of folk religions, plants and other elements of the natural world more evidently conform their idea of the cultural domain *folk religion* than for non-followers.

For the Beninese followers of folk religions, abstract items such as “advantage,” “truth,” “trust,” and “peace” were prevalent notions related to their religious belief. While non-followers provided remarkably detailed lists of Vodoun spirit names and ceremonial objects, they also mentioned several terms that suggested negative values such as “sacrilege,” “sadness,” “violence,” “negativity,” and “immorality,” It is interesting that the non-followers’ detailed knowledge of elements of Vodoun (including plant and animal species) reflects the proximity of this cultural expression, if not lifestyle, to wider sectors of the population other than self-reported followers. These findings corroborate those of Medin and Altran (2004), who, comparing ecological knowledge between novices and experts, found that “standard populations” (i.e., a group of people that is expected to have a certain knowledge) do not necessarily reflect the cognitive consequences of diminished contact with nature—or in this case, of diminished direct exposure to folk religions.

### The Cultural Domain “Bwiti”

In contrast to the results we obtained in Benin, Gabonese, followers of folk religions mentioned elements of the natural world twice as often as non-followers, with plants ranking higher in the Gabonese followers’ ideas of the cultural domain “Bwiti” (Fig. 5). For Gabon, we thus accept our hypothesis that within the conception of their own folk religions, plants are more important to followers than to non-followers of folk religions. Although followers provided almost four times as many terms as non-followers in this category, saliency was comparable in both lists. Similar to our results in Benin, the total non-followers’ list for the cultural domain *folk religion* was characterized by the presence of terms with a negative connotation, such as “ignorance,” “bewitchment,” and “sorcerer.” Whereas the followers’ list also contained such items, these were attributed a lower rank and were cited less frequently. Terms such as “tradition,” God, and “knowledge” were among the most salient in the lists of followers.

As discussed previously, although folk religions are tolerated by a large sector of the Gabonese non-practicing community (US Department of the State 2012), there is still a rather prevalent secrecy around Bwiti—due either to its stigmatization by non-followers or its required confidentiality among followers (Bonhomme 2007). In spite of taking precautions to prevent underreporting, most of the free-listing exercises in Gabon (*n* = 22) were completed by informants unable to read or write (thus, they involved the presence of a second or even a third party to take notes). This could explain the short lists both among followers and non-followers. The Beninese participants, of both urban and rural backgrounds, had a higher education level than those from Gabon. With this bias, it is important to apply caution, as these results might not be transferrable to more diverse populations.

### Plant Species and Products

In total, the informants mentioned eight individual plant species and another 21 plant products or other elements in the plant world, such as “forest” or “sacred grove” (ESM). Gabonese informants provided a larger number of plant species (*n* = 5) than their Beninese counterparts (who only provided 3). Beninese informants, on the other hand, mentioned more plant products (*n* = 7) that the Gabonese ones (who provided only 6). There was an overlap of six plant products or elements of the natural world between the lists of both countries. In Benin, plant products included ceremonial stimulants such as cola nuts (*Cola acuminata* (P.Beauv.) Schott & Endl.) and Guinea pepper (*Aframomum melegueta* K.Schum.), products used in offerings such as African palm oil (*Elaeis guineensis* Jacq.), maize flour (*Zea mays* L.), cowpeas (*Vigna unguiculata* (L.) Walp.), and calabashes (*Lagenaria siceraria* (Molina) Standl., *L. breviflora* (Benth.) Roberty and *Crescentia cujete* L.)*.* Although many ceremonial products can be extracted from the iroko tree (*Milicia excelsa* (Welw.) C.C.Berg), the plant was only named by its vernacular name, and not by its separate parts, probably because the living tree is an important sacred tree throughout West Africa (Ouinsavi et al. 2005). The raffia palm (*Raphia* spp*.*) was the species with the largest variety of products mentioned (i.e., palm wine, leaves, fiber, and seeds, see ESM). This species appeared as a ceremonial plant in the free-lists of both countries, stressing its important ritual role throughout West Africa (Gruca et al. 2014).

Gabonese plant products included red pigment, which is made from the powdered wood of the padouk (*Pterocarpus soyauxii* Taub.) and used ceremonially as a body pigment, the wood of the climbing palm *Eremospatha cabrae* (De Wild. & T. Durand) De Wild. for the elaboration of ceremonial fly whisks, and the bark of the bitter cola (*Garcinia kola* Heckel), a fermentation agent for traditional palm wine. Only adepts in Gabon considered the “flambeau” (a type of torch made from the twigs of *Aframomum giganteum* (Oliv. & D. Hanb.) K. Schum.) and the larger “torche indigène” (the resin of *Aucoumea klaineana* Pierre wrapped in the bark of *Xylopia aethiopica* (Dunal) A.Rich.), as elements of Bwiti. Iboga (*Tabernanthe iboga* Baill.) was mentioned both as a plant product (“bois sacré,” sacred wood) and as a plant species (“eboga”). Other salient plant species linked to Bwiti were the ant tree (*Barteria fistulosa* Mast.), and the alan root (*Alchornea floribunda* Müll.Arg.).

The fact that informants, regardless of their religious affiliation, named the above-mentioned plant products reflects the importance of these species in their social environment. Species such as *A. melegueta*, *C. acuminata*, and *Lagenaria* spp. are sold in large quantities in medicinal plant markets and almost without exception on the ubiquitous roadside stalls of Benin (Quiroz et al. 2014). Likewise, *A. klaineana*, *T. iboga*, *G. kola*, and *P. soyauxii* are among the most frequently sold products in the herbal markets of Gabon (Towns et al. 2014). Many different ritual uses for these species have been documented recently in Benin and Gabon (Quiroz 2015). Apparently, these species are directly associated with folk religion. Considering the definition proposed by Platten and Henfrey (2009), in which cultural keystones species form a complex incorporating several tangible and non-tangible system elements (for example beliefs, ideas, norms, and values concerning social identity and its enactment through culturally appropriate practices), our results confirm those of earlier inventories of ritual plants (Quiroz 2015) that at least *A. klaineana* and *T. iboga* in Gabon and *M. excelsa* in Benin can be considered as cultural keystone species.

### Why Emic Perspectives Matter

Apart from highlighting important aspects of the cultural practices of our countries of study, our results indicate the implications of using the right terminology when conducting research with local informants and asking questions about religion. Far from attempting to engage in an epistemological debate about the definitions of religion or folk religion, or the adequacy of these terms, our results invite a reflection on how informants actually understand these questions and the possible curtailing of research results. By failing to understand the boundaries of a study domain, we risk asking the wrong question for a particular culture (Martin 1995). Interviewing local people on plants used during “religious ceremonies,” for example, might yield very few results as participants can interpret these as activities organized by the church or mosque, which leave little space for spiritual plant use. In their long-term effort to destroy pagan animism, Christianity has been successful in eliminating local beliefs in spirits that inhabited plants, which would ensure their protection (White 1967).

The same can be said about asking informants to provide information about the use of natural resources associated to their magico-religious, or “medico-magical” practices, when they never use these categories themselves. The results from our study suggest that, as long as the emic term for local folk religions is used, Cultural Domain Analysis can be an effective method to document practices where supernatural agency is involved, related plant products, and cultural salient species.

## Conclusions

In our study, we gained understanding of the cultural domains *global religion* and *folk religions* in Benin and Gabon from an emic perspective. We were able to confirm that to the informants, notions of the term (*global*) *religion* reflected a domain more akin to Western than to African cultures. Our findings vindicate the limited role plants play in this domain, both for followers and non-followers of Vodoun and Bwiti. Conversely, plants occupied an important place in people’s conceptions of the folk religions of these two countries, although for the Beninese followers of these faiths, plants did not prove to be more important than for non-followers. This points to the cultural saliency of ritual plants, not only in the context of folk religions but also in the wider, social environments of Benin and Gabon. Assessing the cultural importance of ritual plants in this context could be a starting point to analyze their role as cultural keystone species. Lastly, Cultural Domain Analysis proved to be an effective method to retrieve information on the major ritual plant species and products, as long as the specific local terminology is used with regard to folk religions.

## Electronic Supplementary Material

ESM 1 (DOCX 192 kb)
