# Supplementary material for: The Cultural Importance of Plants in Western African Religions
Source: Econ Bot. 2018 Apr 3;72(3):251–62. doi: 10.1007/s12231-018-9410-x (PMC6267723; doi:10.1007/s12231-018-9410-x)
Supplement: Supplementary file 1 — (DOCX 192 kb) [file 12231_2018_9410_MOESM1_ESM.docx]

Supplementary file. Complete list of terms provided by the 96 informants in Benin and Gabon for the domains “global religion” and “folk religion”.

| Term | Plant species | Elements of plant world or plant products | Domain | | | | | Meaning | | |  |
| --- | --- | --- | --- | --- | --- | --- | --- | --- | --- | --- | --- |
|  |  |  | Global religion | | Folk religion | |  | | |  |  |
|  |  |  | Citation frequency | Average rank | Citation frequency | Average rank | | |  | | |
| Accident |  |  |  |  | 0.01 | 3.00 | | |  | | |
| Advantage |  |  |  |  | 0.01 | 10.00 | | |  | | |
| Adept |  |  | 0.03 | 7.00 | 0.22 | 7.38 | | |  | | |
| Adoration |  |  | 0.06 | 9.50 | 0.08 | 8.63 | | |  | | |
| *Aframomum melegueta* | 1 |  |  |  | 0.02 | 3.50 | | | Large Zingiberaceae herb. Seeds of this plant are used medicinally and ritually throughout Africa. | | |
| Africa |  |  |  |  | 0.01 | 1.00 | | |  | | |
| Air |  |  |  |  | 0.01 | 18.00 | | |  | | |
| Allah |  |  | 0.15 | 7.20 |  |  | | |  | | |
| Albino |  |  |  |  | 0.02 | 3.50 | | |  | | |
| *Alchornea floribunda* [Müll.Arg.](http://www.theplantlist.org/tpl1.1/record/kew-5738) | 1 |  |  |  | 0.02 | 4.50 | | | Euphorbiaceae shrub whose root is used ceremonially to enhance communication with the ancestors. | | |
| Alcoholic beverage |  |  |  |  | 0.07 | 10.43 | | |  | | |
| America |  |  |  |  | 0.01 | 2.00 | | |  | | |
| Ancestor |  |  | 0.05 | 4.67 | 0.06 | 12.00 | | |  | | |
| Ancestral |  |  |  |  | 0.08 | 3.38 | | |  | | |
| Angel |  |  | 0.02 | 3.00 |  |  | | |  | | |
| Animals |  |  | 0.02 | 25.00 | 0.16 | 6.60 | | |  | | |
| Animist |  |  | 0.02 | 16.00 | 0.02 | 14.00 | | |  | | |
| Apocalipsis |  |  | 0.15 | 7.50 |  |  | | |  | | |
| Apostle |  |  | 0.02 | 29.00 |  |  | | |  | | |
| Archbishop |  |  | 0.02 | 16.00 |  |  | | |  | | |
| Asia |  |  |  |  | 0.01 | 3.00 | | |  | | |
| Assemblies of God |  |  | 0.30 | 5.10 |  |  | | | Groups of autonomous churches part of the Pentecostal church. | | |
| Atheist |  |  | 0.03 | 13.50 |  |  | | |  | | |
| Avoid |  |  |  |  | 0.02 | 2.00 | | |  | | |
| Awaken |  |  |  |  | 0.01 | 6.00 | | |  | | |
| Balaphone |  |  | 0.10 | 6.67 | 0.04 | 8.00 | | | Large wooden xylophone (male type). | | |
| Baptism |  |  | 0.05 | 6.00 |  |  | | |  | | |
| *Barteria fistulosa* | 1 |  |  |  | 0.01 | 1.00 | | | Treelet of the Passifloraceae family inhabited by fierce ants (*Tetraponera aethiops*). The tree is used in Bwiti initiation ceremonies to test the courage of men. Known in French as l'arbre de l'adultère (tree of adultery). | | |
| Basket |  |  |  |  | 0.02 | 9.50 | | |  | | |
| Bead |  |  |  |  | 0.04 | 12.75 | | |  | | |
| Beans |  | 1 |  |  | 0.02 | 12.50 | | | Ayikun in the Fon language, or les haricots in French, usually refer to cowpeas (*Vigna unguiculata* (L.) Walp.), a common ceremonial offering and food taboo. | | |
| Behaviour |  |  | 0.02 | 3.00 | 0.02 | 3.50 | | |  | | |
| Believe |  |  | 0.02 | 20.00 | 0.23 | 6.09 | | |  | | |
| Bell |  |  |  |  | 0.02 | 9.00 | | |  | | |
| Benefit |  |  | 0.02 | 7.00 | 0.04 | 11.50 | | |  | | |
| Benediction |  |  |  |  | 0.01 | 24.00 | | |  | | |
| Benefactor |  |  |  |  | 0.01 | 5.00 | | |  | | |
| Benin |  |  |  |  | 0.03 | 18.00 | | |  | | |
| Bewitchment |  |  |  |  | 0.03 | 4.00 | | |  | | |
| Bible |  |  | 0.06 | 2.33 |  |  | | |  | | |
| Birds |  |  |  |  | 0.02 | 4.00 | | |  | | |
| Birth |  |  |  |  | 0.03 | 4.67 | | |  | | |
| Black cloth |  |  |  |  | 0.02 | 14.00 | | |  | | |
| Blood |  |  |  |  | 0.13 | 6.58 | | |  | | |
| Boss |  |  |  |  | 0.01 | 18.00 | | |  | | |
| Buddhism |  |  | 0.02 | 21.00 |  |  | | |  | | |
| Burial |  |  | 0.03 | 12.50 |  |  | | |  | | |
| Burning torch |  | 1 |  |  | 0.10 | 5.50 | | | The flambeau (French) or mododi (Mitsogo) is a torch used in Bwiti ceremonies made from the twigs of *Aframomum giganteum* (Oliv. & D.Hanb.) K.Schum. | | |
| Bwiti |  |  | 0.03 | 10.00 |  |  | | |  | | |
| Bwiti altar |  |  |  |  | 0.04 | 3.67 | | |  | | |
| Calabash |  | 1 |  |  | 0.02 | 9.00 | | | Fruits of *Crescentia cujete* L., *Lagenaria breviflora* (Benth.) Roberty or *L. siceraria* (Molina) Standl. Used as a container for herbal medicine and ceremonial offerings. | | |
| Candle |  |  | 0.02 | 1.00 | 0.04 | 4.50 | | |  | | |
| Caricature |  |  |  |  | 0.01 | 10.00 | | |  | | |
| Cassock |  |  | 0.02 | 7.00 |  |  | | | Ankle-length garment traditionally worn by nuns, monks, and friars. | | |
| Cat |  |  |  |  | 0.02 | 11.00 | | |  | | |
| Catholicism |  |  | 0.02 | 10.00 |  |  | | |  | | |
| Celestial |  |  | 0.02 | 2.00 |  |  | | |  | | |
| Ceremonial food |  |  |  |  | 0.01 | 23.00 | | |  | | |
| Ceremony |  |  |  |  | 0.04 | 10.25 | | |  | | |
| Chant |  |  | 0.02 | 22.00 |  |  | | |  | | |
| Chapel |  |  | 0.06 | 6.75 |  |  | | |  | | |
| Charity |  |  | 0.03 | 8.00 | 0.01 | 9.00 | | |  | | |
| Charlatan |  |  | 0.03 | 3.00 | 0.02 | 2.50 | | | Term carries a negative connotation (cheater or quack), in Benin and Gabon it is the French term with which people refer to traditional healers. | | |
| Chicken |  |  |  |  | 0.08 | 7.50 | | |  | | |
| Child |  |  |  |  | 0.03 | 5.33 | | |  | | |
| Choir |  |  | 0.02 | 15.00 |  |  | | |  | | |
| Christ |  |  | 0.02 | 10.00 |  |  | | |  | | |
| Christianity |  |  | 0.07 | 1.00 | 0.01 | 7.00 | | |  | | |
| Church |  |  | 0.12 | 5.42 |  |  | | |  | | |
| Cicatrices |  |  |  |  | 0.01 | 13.00 | | | Scar. | | |
| City |  |  |  |  | 0.01 | 16.00 | | |  | | |
| Clay |  |  |  |  | 0.03 | 19.00 | | |  | | |
| Clay pot |  |  |  |  | 0.01 | 9.00 | | |  | | |
| Cleanliness |  |  | 0.20 | 6.23 |  |  | | |  | | |
| Cloth |  |  | 0.16 | 6.89 | 0.08 | 10.13 | | |  | | |
| *Cola acuminata* [(P.Beauv.) Schott & Endl.](http://www.theplantlist.org/tpl1.1/record/kew-2730552) | 1 |  |  |  | 0.06 | 8.83 | | | Tree of West and Central African rain forests. Its nuts are eaten as ceremonial stimulants. | | |
| Colonisation |  |  | 0.03 | 4.00 | 0.01 | 22.00 | | |  | | |
| Commandment |  |  | 0.03 | 13.50 | 0.01 | 16.00 | | |  | | |
| Community |  |  | 0.05 | 12.00 | 0.01 | 8.00 | | |  | | |
| Complication |  |  |  |  | 0.01 | 2.00 | | |  | | |
| Communion |  |  | 0.02 | 2.00 |  |  | | |  | | |
| Confession |  |  | 0.06 | 6.25 |  |  | | |  | | |
| Confirmation |  |  | 0.03 | 18.00 |  |  | | |  | | |
| Consecration |  |  | 0.03 | 12.00 |  |  | | |  | | |
| Consultation |  |  |  |  | 0.01 | 6.00 | | |  | | |
| Conversion |  |  | 0.02 | 10.00 |  |  | | |  | | |
| Coran |  |  | 0.02 | 16.00 |  |  | | |  | | |
| Cow |  |  |  |  | 0.03 | 13.00 | | |  | | |
| Cowry |  |  |  |  | 0.04 | 7.25 | | | Ritually used shells, often belonging to the species *Monetaria moneta.* | | |
| Creation |  |  |  |  | 0.01 | 9.00 | | |  | | |
| Cross |  |  | 0.02 | 11.00 | 0.01 | 13.00 | | |  | | |
| Crucifixion |  |  | 0.03 | 14.00 |  |  | | |  | | |
| Cult |  |  | 0.02 | 13.00 | 0.08 | 5.75 | | |  | | |
| Culture |  |  | 0.04 | 11.00 | 0.05 | 7.00 | | |  | | |
| Cure |  |  |  |  | 0.03 | 9.33 | | |  | | |
| Custom |  |  | 0.09 | 7.50 |  |  | | |  | | |
| Dah |  |  |  |  | 0.03 | 7.67 | | | Ancient king of Benin, origin of the word Dahomey (Benin's former official name) is said to be derived, meaning ‘the abdomen of Dah’. | | |
| Dan |  |  | 0.06 | 10.00 | 0.07 | 7.00 | | | God of the Pedah tribes in southern Benin, which is represented by a (Python) serpent. | | |
| Dance |  |  | 0.05 | 7.25 | 0.20 | 8.00 | | |  | | |
| Danger |  |  |  |  | 0.01 | 3.00 | | |  | | |
| Deacon |  |  | 0.03 | 13.00 |  |  | | | An ordained minister of an order ranking below that of priest. | | |
| Death |  |  | 0.02 | 10.00 | 0.13 | 9.33 | | |  | | |
| Demonisation |  |  |  |  | 0.01 | 17.00 | | |  | | |
| Destiny |  |  |  |  | 0.01 | 10.00 | | |  | | |
| Development |  |  |  |  | 0.02 | 15.00 | | |  | | |
| Devil |  |  | 0.08 | 9.00 | 0.05 | 9.60 | | |  | | |
| Dignitary |  |  | 0.03 | 24.50 |  |  | | | A person considered to be important because of high rank or office | | |
| Disciple |  |  |  |  | 0.01 | 5.00 | | |  | | |
| Disease |  |  |  |  | 0.05 | 10.40 | | |  | | |
| Disown |  |  |  |  | 0.01 | 2.00 | | |  | | |
| Divinity |  |  | 0.02 | 1.00 | 0.19 | 6.39 | | |  | | |
| Doubt |  |  |  |  | 0.01 | 21.00 | | |  | | |
| Dress code |  |  |  |  | 0.01 | 14.00 | | |  | | |
| Drums |  |  | 0.10 | 5.67 | 0.09 | 5.89 | | |  | | |
| Earth |  |  | 0.05 | 14.00 | 0.02 | 9.00 | | |  | | |
| Eckankar |  |  | 0.02 | 7.00 |  |  | | | Religious movement founded by Paul Twitchell in 1965. | | |
| Ecumenical |  |  | 0.02 | 17.00 |  |  | | |  | | |
| Education |  |  | 0.02 | 2.00 | 0.01 | 3.00 | | |  | | |
| Effect |  |  |  |  | 0.02 | 6.00 | | |  | | |
| Egg |  |  |  |  | 0.01 | 2.00 | | |  | | |
| Egougou |  |  |  |  | 0.01 | 2.00 | | | The word refers to the Yoruba masquerades connected with ancestor worship. | | |
| Egon |  |  |  |  | 0.06 | 10.00 | | | Yoruba term for the living dead, represented by special costumes in southern Benin. | | |
| Egu |  |  |  |  | 0.01 | 10.00 | | | God of iron and war in the Vodoun pantheon in Benin. | | |
| Elaborate |  |  |  |  | 0.01 | 1.00 | | |  | | |
| Elders |  |  | 0.06 | 12.00 |  |  | | |  | | |
| Elements |  |  |  |  | 0.01 | 10.00 | | |  | | |
| End |  |  | 0.02 | 6.00 |  |  | | |  | | |
| Equity |  |  | 0.02 | 7.00 |  |  | | |  | | |
| Escape |  |  |  |  | 0.01 | 2.00 | | |  | | |
| Essence |  |  |  |  | 0.01 | 1.00 | | | The intrinsic nature of things. | | |
| Eternal life |  |  | 0.02 | 5.00 |  |  | | |  | | |
| Europe |  |  |  |  | 0.01 | 4.00 | | |  | | |
| Evangelism |  |  | 0.03 | 13.50 |  |  | | |  | | |
| Evil |  |  | 0.08 | 12.00 | 0.08 | 7.00 | | |  | | |
| Evil spirit |  |  | 0.03 | 3.00 |  |  | | |  | | |
| Evolution |  |  | 0.03 | 13.00 |  |  | | |  | | |
| Existence |  |  |  |  | 0.02 | 4.00 | | |  | | |
| Fâ |  |  | 0.02 | 12.00 | 0.06 | 13.50 | | | Divinatory science of Benin and Nigeria. | | |
| Faith |  |  | 0.03 | 6.75 | 0.04 | 7.50 | | |  | | |
| Faithfulness |  |  | 0.27 | 6.22 |  |  | | |  | | |
| Falsehood |  |  |  |  | 0.01 | 8.00 | | |  | | |
| Family |  |  | 0.02 | 22.00 | 0.04 | 7.50 | | |  | | |
| Fang |  |  |  |  | 0.03 | 7.33 | | | Ethnic group of Gabon, Cameroun, and Equatorial Guinea. | | |
| Father |  |  |  |  | 0.01 | 10.00 | | |  | | |
| Fatigue |  |  | 0.03 | 4.50 |  |  | | |  | | |
| Fear |  |  |  |  | 0.02 | 12.50 | | |  | | |
| Feast |  |  |  |  | 0.02 | 9.00 | | |  | | |
| Feather |  |  |  |  | 0.01 | 22.00 | | |  | | |
| Feed |  |  |  |  | 0.03 | 4.33 | | | Offering food to the spirits. | | |
| Female drum |  |  |  |  | 0.01 | 9.00 | | |  | | |
| Fetish |  |  |  |  | 0.08 | 5.50 | | |  | | |
| Fetish priest |  |  | 0.02 | 9.00 | 0.07 | 5.43 | | |  | | |
| Fire |  |  |  |  | 0.02 | 14.50 | | |  | | |
| Fly-whisk |  | 1 |  |  | 0.02 | 8.00 | | | Ceremonial object of Fâ oracle priests in Benin and male Bwiti initiates in Gabon. In Gabon, it is made from the rattan *Eremospatha cabrae* (De Wild. & T.Durand) De Wild, while in Benin usually from horse tails. | | |
| Force |  |  |  |  | 0.01 | 4.00 | | |  | | |
| Forest |  | 1 | 0.07 | 2.00 | 0.02 | 13.00 | | |  | | |
| Forest spirit |  |  |  |  | 0.02 | 5.50 | | |  | | |
| Forgiveness |  |  | 0.05 | 9.38 |  |  | | |  | | |
| France |  |  | 0.03 | 3.00 |  |  | | |  | | |
| Free Mason |  |  | 0.15 | 6.60 |  |  | | |  | | |
| Freedom |  |  | 0.03 | 7.00 |  |  | | |  | | |
| Fruit |  | 1 |  |  | 0.01 | 19.00 | | |  | | |
| Gambara |  |  |  |  | 0.02 | 5.50 | | | Type of Vodoun ceremony from Allada, Benin. | | |
| *Garcinia kola* Heckel bark | 1 |  |  |  | 0.01 | 12.00 | | | Tree of the Clusiaceae family whose bark is used to make palm wine in Gabon. Its seeds are used ceremonially as stimulants. | | |
| Girl |  |  |  |  | 0.01 | 9.00 | | |  | | |
| Glory |  |  | 0.05 | 11.33 |  |  | | |  | | |
| Goat |  |  |  |  | 0.01 | 17.00 | | |  | | |
| God |  |  | 0.08 | 7.75 | 0.17 | 5.44 | | |  | | |
| Gods |  |  |  |  | 0.04 | 2.50 | | |  | | |
| Good |  |  | 0.03 | 11.00 | 0.08 | 6.25 | | |  | | |
| Grace |  |  | 0.18 | 2.67 | 0.01 | 12.00 | | |  | | |
| Gris-gris |  |  |  |  | 0.03 | 13.00 | | | Magical object or charm. | | |
| Gueledé |  |  |  |  | 0.01 | 9.00 | | | Type of masquerades of southern Benin connected with ancestor worship. | | |
| Hand |  |  |  |  | 0.01 | 2.00 | | |  | | |
| Happiness |  |  |  |  | 0.04 | 4.00 | | |  | | |
| Harp |  |  | 0.03 | 2.00 | 0.03 | 10.33 | | | Ceremonial string instrument played by the mouth during Bwiti ceremonies, called Mongongo (male) or Ngombi (female). | | |
| Healer |  |  | 0.02 | 11.00 | 0.03 | 12.33 | | |  | | |
| Health |  |  |  |  | 0.01 | 4.00 | | |  | | |
| Heart |  |  | 0.02 | 6.00 |  |  | | |  | | |
| Hell |  |  | 0.02 | 4.00 | 0.01 | 6.00 | | |  | | |
| Herbal bath |  | 1 |  |  | 0.01 | 5.00 | | |  | | |
| Hêviosso |  |  |  |  | 0.16 | 5.27 | | | God of thunder in Benin. | | |
| Hideout |  |  |  |  | 0.01 | 3.00 | | |  | | |
| History |  |  | 0.02 | 10.00 |  |  | | |  | | |
| Holy ghost |  |  | 0.02 | 10.00 |  |  | | |  | | |
| Horn |  |  |  |  | 0.03 | 9.67 | | | Ceremonial instrument made from the horn of a cow used in Bwiti vigils. | | |
| Human |  |  | 0.09 | 7.50 |  |  | | |  | | |
| Human skull |  |  |  |  | 0.02 | 12.50 | | |  | | |
| Humanity |  |  |  |  | 0.14 | 6.46 | | |  | | |
| Ideology |  |  | 0.06 | 8.75 |  |  | | |  | | |
| Ignorance |  |  |  |  | 0.02 | 5.00 | | |  | | |
| Incantation |  |  |  |  | 0.09 | 11.67 | | |  | | |
| Individual |  |  | 0.02 | 10.00 |  |  | | |  | | |
| Initiation |  |  |  |  | 0.08 | 8.75 | | |  | | |
| Initiates |  |  |  |  | 0.02 | 11.50 | | |  | | |
| Injure |  |  | 0.05 | 3.00 |  |  | | |  | | |
| Imam |  |  | 0.02 | 8.00 |  |  | | |  | | |
| Immorality |  |  |  |  | 0.01 | 6.00 | | |  | | |
| Impossibility |  |  | 0.08 | 11.60 |  |  | | |  | | |
| Incence |  |  | 0.02 | 3.00 |  |  | | |  | | |
| Instruments |  |  | 0.05 | 5.33 | 0.01 | 10.00 | | |  | | |
| Intelligence |  |  |  |  | 0.01 | 12.00 | | |  | | |
| Invisible |  |  |  |  | 0.01 | 7.00 | | |  | | |
| Invisible being |  |  | 0.02 | 9.00 |  |  | | |  | | |
| Iroko | 1 |  |  |  | 0.01 | 21.00 | | | Large forest tree (*Milicia excelsa* (Welw.) C.C.Berg) of West and Central Africa. Considered as sacred in Benin | | |
| Iron |  |  |  |  | 0.03 | 4.00 | | |  | | |
| Islam |  |  | 0.04 | 8.00 |  |  | | |  | | |
| Iya-Agan |  |  |  |  | 0.01 | 8.00 | | | In the Yoruba Egugun mythology, the foster mother of Agan (a half-human, half-monkey child). | | |
| Jehova |  |  | 0.32 | 7.57 |  |  | | |  | | |
| Jerusalem |  |  | 0.05 | 8.33 |  |  | | |  | | |
| Jesus |  |  | 0.02 | 17.00 |  |  | | |  | | |
| Joy |  |  |  |  | 0.01 | 12.00 | | |  | | |
| Judaism |  |  | 0.14 | 5.33 |  |  | | |  | | |
| Judgement day |  |  | 0.03 | 12.50 |  |  | | |  | | |
| Kaolin |  |  |  |  | 0.01 | 9.00 | | |  | | |
| Kétou |  |  |  |  | 0.01 | 10.00 | | | City in the Plateau department of Benin. | | |
| King |  |  |  |  | 0.02 | 8.50 | | |  | | |
| Knowledge |  |  |  |  | 0.06 | 7.83 | | |  | | |
| Kôkou |  |  |  |  | 0.02 | 5.50 | | | A feared warrior under god of the Yoruba. Also, a town in Benin. | | |
| Konfo |  |  |  |  | 0.01 | 10.00 | | | Village in Benin. | | |
| Lamb |  |  |  |  | 0.02 | 13.00 | | |  | | |
| Language |  |  | 0.02 | 10.00 |  |  | | |  | | |
| Law of nature |  |  | 0.02 | 8.00 | 0.01 | 8.00 | | |  | | |
| Lesser god |  |  |  |  | 0.02 | 3.50 | | |  | | |
| Legba |  |  |  |  | 0.16 | 3.00 | | | God of chaos, crossroads and protector of homes in Benin. | | |
| Life |  |  | 0.03 | 8.00 | 0.03 | 5.67 | | |  | | |
| Lie |  |  | 0.02 | 18.00 |  |  | | |  | | |
| Light |  |  |  |  | 0.01 | 1.00 | | |  | | |
| Lightning |  |  |  |  | 0.01 | 4.00 | | |  | | |
| Lisa |  |  |  |  | 0.01 | 5.00 | | | The masculine aspect of the androgynous creator god Mawu-Lisa in the Vodoun pantheon. | | |
| Listen |  |  | 0.06 | 12.25 | 0.01 | 18.00 | | |  | | |
| Lodge |  |  | 0.02 | 1.00 |  |  | | |  | | |
| Longevity |  |  | 0.02 | 7.00 |  |  | | |  | | |
| Love |  |  | 0.02 | 3.00 | 0.02 | 5.50 | | |  | | |
| Lucifer |  |  | 0.17 | 5.00 | 0.01 | 10.00 | | | Another name for Satan. | | |
| Maintenance |  |  | 0.02 | 19.00 |  |  | | |  | | |
| Maize flour |  | 1 |  |  | 0.10 | 7.40 | | | Flour of *Zea mays* L. is largely used ceremonially in Benin. | | |
| M'alan |  |  |  |  | 0.01 | 5.00 | | | Initiatic society of the Fang in Gabon. Adepts of this traditional faith ceremonially consume the roots of *Alchornea floribunda* [Müll.Arg.](http://www.theplantlist.org/tpl1.1/record/kew-5738). The plant is known as Alan (Fang), thus also the name of the traditional faith. | | |
| Male drum |  |  |  |  | 0.01 | 7.00 | | | Called Nomnkoul. | | |
| Malefactor |  |  |  |  | 0.01 | 4.00 | | |  | | |
| Mami Wata |  |  |  |  | 0.09 | 5.44 | | | Goddess of water in West and Central Africa. | | |
| Man |  |  |  |  | 0.02 | 9.00 | | |  | | |
| Mao-Tse-Tung |  |  | 0.02 | 10.00 |  |  | | |  | | |
| Marriage |  |  | 0.02 | 13.00 | 0.01 | 15.00 | | |  | | |
| Mat |  | 1 |  |  | 0.01 | 3.00 | | | Woven mats of *Raphia* fibre. | | |
| Matthew |  |  | 0.03 | 5.00 |  |  | | |  | | |
| Mecca |  |  | 0.02 | 16.00 |  |  | | |  | | |
| Medicinal plants |  | 1 | 0.02 | 2.00 | 0.01 | 2.00 | | | In French, referred to as ‘les feuilles’. | | |
| Medicine |  |  |  |  | 0.01 | 24.00 | | |  | | |
| Meditation |  |  |  |  | 0.01 | 2.00 | | |  | | |
| Mercy |  |  | 0.02 | 12.00 |  |  | | |  | | |
| Messenger |  |  |  |  | 0.01 | 13.00 | | |  | | |
| Methodist |  |  | 0.02 | 4.00 |  |  | | |  | | |
| Missoko |  |  |  |  | 0.01 | 3.00 | | | A branch of Bwiti exclusive for traditional healers. | | |
| Mitsogo |  |  |  |  | 0.01 | 1.00 | | | Ethnic group of Gabon. | | |
| Mohammed |  |  | 0.02 | 23.00 |  |  | | |  | | |
| Monastery |  |  | 0.05 | 9.67 |  |  | | |  | | |
| Money |  |  | 0.02 | 15.00 | 0.03 | 6.67 | | |  | | |
| Moon |  |  |  |  | 0.02 | 8.00 | | |  | | |
| Mosque |  |  | 0.03 | 7.50 |  |  | | |  | | |
| Mother |  |  |  |  | 0.01 | 11.00 | | |  | | |
| Music |  |  |  |  | 0.01 | 9.00 | | |  | | |
| Mutton |  |  | 0.08 | 11.40 |  |  | | | Flesh of mature sheep. | | |
| Mwiri |  |  |  |  | 0.02 | 4.50 | | | Male secret society of Gabon, a branch of Bwiti. | | |
| Nature |  |  |  |  | 0.06 | 9.33 | | |  | | |
| Needle |  |  |  |  | 0.01 | 13.00 | | |  | | |
| Negativity |  |  |  |  | 0.01 | 7.00 | | |  | | |
| Neighbour |  |  | 0.02 | 9.00 |  |  | | |  | | |
| Nganga |  |  |  |  | 0.22 | 4.43 | | | In Gabon, the name of Bwiti spiritual guide. | | |
| Night |  |  |  |  | 0.11 | 7.45 | | |  | | |
| Nun |  |  | 0.03 | 7.00 |  |  | | |  | | |
| Nyembe |  |  |  |  | 0.01 | 3.00 | | | Female secret society of Gabon. | | |
| Offering |  |  | 0.02 | 9.00 | 0.06 | 9.67 | | |  | | |
| Ogboni |  |  | 0.02 | 6.00 |  |  | | | Fraternal institution indigenous to the Yoruba language-speaking polities of Nigeria, Republic of Benin and Togo. | | |
| Ogoun |  |  |  |  | 0.01 | 4.00 | | | Yoruba god of iron. | | |
| Open |  |  |  |  | 0.01 | 20.00 | | |  | | |
| Opium |  |  | 0.02 | 18.00 |  |  | | |  | | |
| Oracle |  |  |  |  | 0.01 | 10.00 | | |  | | |
| Oro |  |  |  |  | 0.02 | 13.50 | | | Female spirit of wind in the Egugun mythology of the Yoruba, a male-only cult in Benin. | | |
| Ouidah |  |  |  |  | 0.03 | 21.33 | | | Coastal city of Benin. | | |
| Our lady |  |  | 0.02 | 11.00 |  |  | | |  | | |
| Owl |  |  |  |  | 0.02 | 16.00 | | |  | | |
| Pact |  |  | 0.02 | 11.00 | 0.01 | 6.00 | | |  | | |
| Paedophilia |  |  | 0.06 | 11.25 |  |  | | |  | | |
| Paradise |  |  | 0.02 | 8.00 | 0.01 | 7.00 | | |  | | |
| Parrot feather |  |  |  |  | 0.05 | 9.00 | | | Both in Benin and Gabon, the red tail feathers of the African grey parrot (*Psittacus erithacus*) are worn in initiation ceremonies. | | |
| Passion |  |  | 0.05 | 16.00 |  |  | | |  | | |
| Pastor |  |  | 0.02 | 4.00 |  |  | | |  | | |
| Pate rouge |  | 1 |  |  | 0.01 | 4.00 | | | Traditional dish of Benin made of maize flour and tomato and a common ceremonial offering. Locally known as Amiwô (Fon). | | |
| Path |  |  | 0.02 | 5.00 |  |  | | |  | | |
| Peace |  |  | 0.03 | 8.00 | 0.01 | 5.00 | | |  | | |
| Pentecostal |  |  | 0.02 | 7.00 |  |  | | |  | | |
| Perfume |  |  |  |  | 0.04 | 3.75 | | |  | | |
| Peter |  |  | 0.03 | 6.50 |  |  | | |  | | |
| Phenomenon |  |  |  |  | 0.01 | 4.00 | | |  | | |
| Physical life |  |  | 0.02 | 15.00 |  |  | | |  | | |
| Pig |  |  |  |  | 0.01 | 19.00 | | |  | | |
| Plants |  | 1 | 0.02 | 2.00 | 0.21 | 6.55 | | |  | | |
| Platform |  |  | 0.03 | 1.50 |  |  | | |  | | |
| Pobé |  |  |  |  | 0.01 | 11.00 | | | Town of the Ouemé valley in Benin. | | |
| Pommade |  | 1 |  |  | 0.01 | 2.00 | | | Cream mixed with plant extracts used to protect against evil spirits. | | |
| Pope |  |  | 0.02 | 20.00 |  |  | | |  | | |
| Positivity |  |  | 0.06 | 7.75 |  |  | | |  | | |
| Possibility |  |  | 0.02 | 12.00 |  |  | | |  | | |
| Powder |  |  |  |  | 0.01 | 12.00 | | |  | | |
| Power |  |  |  |  | 0.02 | 7.50 | | |  | | |
| Powerlessness |  |  |  |  | 0.01 | 2.00 | | |  | | |
| Practice |  |  | 0.02 | 16.00 | 0.02 | 5.00 | | |  | | |
| Praise |  |  | 0.02 | 4.00 |  |  | | |  | | |
| Prayer |  |  | 0.02 | 5.00 | 0.01 | 5.00 | | |  | | |
| Prescription |  |  |  |  | 0.01 | 11.00 | | |  | | |
| Priest |  |  | 0.08 | 4.33 | 0.04 | 12.50 | | |  | | |
| Principle |  |  |  |  | 0.01 | 23.00 | | |  | | |
| Problem |  |  | 0.21 | 7.00 |  |  | | |  | | |
| Promise |  |  |  |  | 0.01 | 9.00 | | |  | | |
| Prophet |  |  | 0.02 | 3.00 | 0.01 | 14.00 | | |  | | |
| Protect |  |  | 0.02 | 6.00 |  |  | | |  | | |
| Protection |  |  |  |  | 0.06 | 7.33 | | |  | | |
| Protestant |  |  | 0.03 | 8.50 |  |  | | |  | | |
| Psalm |  |  | 0.09 | 11.83 |  |  | | |  | | |
| Pygmies |  |  |  |  | 0.04 | 7.25 | | |  | | |
| Queen |  |  |  |  | 0.02 | 7.00 | | | Wife of an African traditional King. | | |
| Quietness |  |  | 0.02 | 7.00 |  |  | | |  | | |
| Rainbow |  |  |  |  | 0.02 | 5.50 | | |  | | |
| Ram |  |  |  |  | 0.02 | 9.50 | | | Male goat. | | |
| *Raphia* spp. | 1 |  | 0.03 | 4.00 | 0.04 | 10.25 | | | Genus of the family Arecaceae. | | |
| *Raphia* cloth |  | 1 |  |  | 0.01 | 20.00 | | | Traditional cloth made of young leaves of *Raphia* palms | | |
| *Raphia* (palm) leaves |  | 1 | 0.07 | 7.50 | 0.02 | 9.17 | | | The young leaves of the *Raphia* palm, used in a large number of ceremonies (traditional or otherwise). | | |
| Rattle |  |  | 0.03 | 3.00 | 0.01 | 9.00 | | |  | | |
| Rebirth |  |  | 0.02 | 10.00 |  |  | | |  | | |
| Red kaolin |  | 1 | 0.03 | 9.00 | 0.05 | 11.60 | | | Ceremonial pigment. In Benin made from the powdered roots of *Baphia nitida* Lodd. or red clay. In Gabon, made from the powdered wood of *Pterocarpus soyauxii* Taub. or, occasionally, the seeds of *Bixa orellana* L. | | |
| Red cloth |  |  | 0.03 | 9.00 | 0.06 | 10.17 | | |  | | |
| Redemption |  |  | 0.07 | 7.00 |  |  | | |  | | |
| Red oil |  | 1 |  |  | 0.20 | 8.47 | | | Oil extracted from the palm *Elaeis guineensis* Jacq., largely used for food, medicine, and spirituality. | | |
| Refusal |  |  |  |  | 0.01 | 6.00 | | |  | | |
| Religion |  |  |  |  | 0.13 | 4.50 | | |  | | |
| Remedy |  |  |  |  | 0.01 | 10.00 | | |  | | |
| Request |  |  |  |  | 0.03 | 4.67 | | |  | | |
| Respect |  |  | 0.03 | 2.00 | 0.06 | 7.00 | | |  | | |
| Resurrection |  |  | 0.06 | 4.00 |  |  | | |  | | |
| Revelation |  |  | 0.03 | 16.50 |  |  | | |  | | |
| Revenant |  |  |  |  | 0.04 | 7.50 | | | Person who has returned from death (general term). | | |
| Ritual |  |  | 0.02 | 4.00 | 0.03 | 10.33 | | |  | | |
| Rivalry |  |  |  |  | 0.01 | 16.00 | | |  | | |
| River |  |  |  |  | 0.01 | 13.00 | | |  | | |
| Sacred grove |  | 1 |  |  | 0.02 | 4.00 | | |  | | |
| Sacrifice |  |  | 0.02 | 11.00 | 0.15 | 8.93 | | |  | | |
| Sacrilege |  |  |  |  | 0.01 | 13.00 | | |  | | |
| Sadness |  |  |  |  | 0.01 | 4.00 | | |  | | |
| Saints |  |  | 0.02 | 7.00 |  |  | | |  | | |
| Saint sacrament |  |  | 0.02 | 10.00 |  |  | | |  | | |
| Sakpata |  |  | 0.02 | 4.00 | 0.25 | 4.46 | | | Vodoun god of earth and smallpox in Benin. | | |
| Sand |  |  |  |  | 0.03 | 3.33 | | |  | | |
| Satan |  |  | 0.02 | 8.00 | 0.03 | 8.00 | | |  | | |
| Science |  |  | 0.02 | 17.00 | 0.01 | 4.00 | | |  | | |
| Sea |  |  |  |  | 0.01 | 17.00 | | |  | | |
| Sect |  |  | 0.02 | 2.00 | 0.01 | 1.00 | | |  | | |
| See |  |  |  |  | 0.01 | 1.00 | | |  | | |
| Self |  |  | 0.02 | 1.00 |  |  | | |  | | |
| Seminary |  |  | 0.02 | 5.00 |  |  | | |  | | |
| Serpent |  |  |  |  | 0.02 | 9.50 | | |  | | |
| Share |  |  | 0.02 | 17.00 | 0.01 | 12.00 | | |  | | |
| Sheep |  |  |  |  | 0.02 | 9.50 | | |  | | |
| Simplicity |  |  |  |  | 0.01 | 1.00 | | |  | | |
| Sin |  |  | 0.02 | 4.00 |  |  | | |  | | |
| Sitar |  |  | 0.03 | 2.00 | 0.05 | 8.00 | | | Musical instrument played in Bwiti ceremonies. | | |
| Sitar player |  |  |  |  | 0.01 | 5.00 | | |  | | |
| Small balaphone |  |  |  |  | 0.01 | 8.00 | | | Small wooden xylophone (female version). | | |
| Soap |  |  |  |  | 0.02 | 3.50 | | |  | | |
| Sodabi |  | 1 |  |  | 0.01 | 5.00 | | | Alcoholic beverage of Benin made from the fermented sap of the African oil palm (*Elaeis guineensis* Jacq.). | | |
| Solution |  |  | 0.06 | 6.00 |  |  | | |  | | |
| Song |  |  | 0.07 | 8.00 | 0.03 | 14.33 | | |  | | |
| Sorcerer |  |  |  |  | 0.05 | 6.20 | | |  | | |
| Sorcery |  |  |  |  | 0.07 | 9.29 | | |  | | |
| Soul |  |  | 0.02 | 10.00 |  |  | | |  | | |
| Species |  |  |  |  | 0.01 | 6.00 | | | Creature. | | |
| Spirit |  |  | 0.02 | 20.00 | 0.25 | 5.54 | | |  | | |
| Spirituality |  |  |  |  | 0.03 | 8.00 | | |  | | |
| Star |  |  |  |  | 0.05 | 7.00 | | |  | | |
| Statuette |  |  | 0.11 | 10.14 | 0.02 | 6.00 | | | Small statue or figurine commonly made of wood. | | |
| Subjugation |  |  | 0.02 | 6.00 |  |  | | |  | | |
| Submission |  |  | 0.02 | 8.00 | 0.01 | 13.00 | | |  | | |
| Sun |  |  |  |  | 0.02 | 7.00 | | |  | | |
| Symbol |  |  |  |  | 0.01 | 12.00 | | |  | | |
| *Tabernanthe iboga* Baill. | 1 |  |  |  | 0.20 | 4.63 | | | Shrub of the Apocynaceae family. Sacred and medicinal plant of Gabon used in Bwiti ceremonies. | | |
| Taboo |  |  |  |  | 0.02 | 12.00 | | |  | | |
| Tam-tam |  |  |  |  | 0.02 | 15.00 | | | Traditional drum. | | |
| Tchakatou |  |  |  |  | 0.02 | 12.00 | | | Common term for witchcraft or bewitchment in Benin | | |
| Teaching |  |  |  |  | 0.01 | 2.00 | | |  | | |
| Temple |  |  | 0.09 | 4.40 | 0.01 | 21.00 | | |  | | |
| Terror |  |  | 0.02 | 9.00 |  |  | | |  | | |
| Testament |  |  | 0.02 | 3.00 |  |  | | |  | | |
| Thread |  |  |  |  | 0.03 | 6.33 | | |  | | |
| Thrôn |  |  |  |  | 0.11 | 8.27 | | | A recently introduced Vodoun in Benin, said to be originally a god of the Peul tribes in Northern Ghana and protector of those suffering from physical and spiritual disequilibria. | | |
| Thunder |  |  |  |  | 0.03 | 11.67 | | |  | | |
| Tolerance |  |  | 0.02 | 8.00 | 0.01 | 14.00 | | |  | | |
| Totem |  |  |  |  | 0.02 | 18.50 | | | Element of the natural world (usually an animal) adopted as emblem by particular societies or tribes because of the spiritual significance attributed to it. | | |
| Tôtôssou |  |  |  |  | 0.02 | 8.00 | | | Village in Benin. | | |
| Tradition |  |  | 0.03 | 10.50 | 0.10 | 4.00 | | |  | | |
| Traditional torch |  | 1 |  |  | 0.10 | 5.33 | | | Large ceremonial torch made from the resin of *Aucoumea klaineana* and the bark of *Xylopia aethiopica.* | | |
| Trees |  | 1 |  |  | 0.02 | 12.00 | | |  | | |
| Trust |  |  | 0.03 | 13.50 | 0.01 | 6.00 | | |  | | |
| Truth |  |  | 0.03 | 10.50 | 0.01 | 9.00 | | |  | | |
| Vampire |  |  |  |  | 0.04 | 4.00 | | | In Gabon, the term is commonly used to refer to people who, possessed by evil spirits, do evil to others. | | |
| Verse |  |  | 0.06 | 9.25 |  |  | | |  | | |
| Vigil |  |  |  |  | 0.13 | 7.17 | | | La veillé, in French, a ceremony that takes place at night. | | |
| Village |  |  | 0.02 | 11.00 | 0.01 | 17.00 | | |  | | |
| Violence |  |  |  |  | 0.01 | 6.00 | | |  | | |
| Virgin Mary |  |  | 0.02 | 1.00 |  |  | | |  | | |
| Vodoun |  |  | 0.11 | 7.86 |  |  | | |  | | |
| Vodoun child |  |  |  |  | 0.06 | 9.33 | | | Any adept of Vodoun (adults and children). | | |
| Voice |  |  |  |  | 0.01 | 3.00 | | |  | | |
| War |  |  | 0.30 | 6.70 |  |  | | |  | | |
| Wash |  |  |  |  | 0.01 | 5.00 | | |  | | |
| Water |  |  | 0.02 | 1.00 | 0.03 | 10.00 | | |  | | |
| West |  |  |  |  | 0.01 | 21.00 | | |  | | |
| White cloth |  |  |  |  | 0.02 | 13.50 | | |  | | |
| White kaolin |  |  | 0.03 | 8.00 | 0.05 | 9.80 | | | Ceremonial pigment made of white clay. | | |
| Wisdom |  |  | 0.05 | 5.67 |  |  | | |  | | |
| Women |  |  | 0.02 | 13.00 | 0.01 | 3.00 | | |  | | |
| Word |  |  | 0.02 | 13.00 | 0.05 | 6.80 | | |  | | |
| World |  |  |  |  | 0.01 | 6.00 | | |  | | |
| Work |  |  | 0.05 | 4.00 |  |  | | |  | | |
| Zangbêto |  |  |  |  | 0.06 | 12.00 | | | Traditional ‘night watchmen’ (nocturnal spirits) of Benin and Nigeria. | | |
| 10 January |  |  |  |  | 0.01 | 5.00 | | | Day of the year when traditional faiths are officially celebrated in Benin. | | |
